# Leisure-Time Physical Activity and Falls With and Without Injuries Among Older Adult Women

**DOI:** 10.1001/jamanetworkopen.2023.54036

**Published:** 2024-01-31

**Authors:** Wing S. Kwok, Saman Khalatbari-Soltani, Xenia Dolja-Gore, Julie Byles, Anne Tiedemann, Marina B. Pinheiro, Juliana S. Oliveira, Catherine Sherrington

**Affiliations:** 1 Sydney Musculoskeletal Health, The University of Sydney, Sydney Local Health District and Northern Sydney Local Health District, Sydney, New South Wales, Australia; 2 Institute for Musculoskeletal Health, The University of Sydney and Sydney Local Health District, Sydney, New South Wales, Australia; 3 School of Public Health, Faculty of Medicine and Health, The University of Sydney, New South Wales, Australia; 4 ARC Centre of Excellence in Population Aging Research (CEPAR), The University of Sydney, Sydney, New South Wales, Australia; 5 School of Medicine and Public Health, The University of Newcastle, Callaghan, New South Wales, Australia

## Abstract

**Importance:**

Falls and fall-related injuries are common among older adults. Older adults are recommended to undertake 150 to 300 minutes of physical activity per week for health benefits; however, the association between meeting the recommended level of physical activity and falls is unclear.

**Objectives:**

To examine whether associations exist between leisure-time physical activity and noninjurious and injurious falls in older women.

**Design, Setting, and Participants:**

This population-based cohort study used a retrospective analysis of the Australian Longitudinal Study on Women’s Health (ALSWH). ALSWH participants born from 1946 to 1951 who completed follow-up questionnaires in 2016 (aged 65-70 years) and 2019 (aged 68-73 years) were included. Statistical analysis was performed from September 2022 to February 2023.

**Exposure:**

Self-reported weekly amounts (0, 1 to <150, 150 to <300, ≥300 minutes) and types of leisure-time physical activity, including brisk walking and moderate- and vigorous-intensity physical activity, in the 2016 survey.

**Main outcome and measures:**

Noninjurious and injurious falls in the previous 12 months reported in the 2019 survey. Associations between leisure-time physical activity and falls were quantified using directed acyclic graph–informed multinomial logistic regression and presented in odds ratios (ORs) and 95% CIs.

**Results:**

This study included 7139 women (mean [SD] age, 67.7 [1.5] years). Participation in leisure-time physical activity at or above the level recommended by the World Health Organization (150 to <300 min/wk) was associated with reduced odds of noninjurious falls (150 to <300 min/wk: OR, 0.74 [95% CI, 0.59-0.92]; ≥300 min/wk: OR, 0.66 [95% CI, 0.54-0.80]) and injurious falls (150 to <300 min/wk: OR, 0.70 [95% CI, 0.56-0.88]; ≥300 min/wk: OR, 0.77 [95% CI, 0.63-0.93]). Compared with women who reported no leisure-time physical activity, those who reported brisk walking (OR, 0.83 [95% CI, 0.70-0.97]), moderate leisure-time physical activity (OR, 0.81 [95% CI, 0.70-0.93]), or moderate-vigorous leisure-time physical activity (OR, 0.84 [95% CI, 0.70-0.99]) had reduced odds of noninjurious falls. No statistically significant associations were found between the types of leisure-time physical activity and injurious falls.

**Conclusions and Relevance:**

Participation in leisure-time physical activity at the recommended level or above was associated with lower odds of both noninjurious and injurious falls. Brisk walking and both moderate and moderate-vigorous leisure-time physical activity were associated with lower odds of noninjurious falls.

## Key Points

**Question:**

Are there associations between leisure-time physical activity and falls with and without injuries among older women?

**Findings:**

This cohort study of 7139 older women found that participation in leisure-time physical activity at the recommended level by the World Health Organization (150 to <300 min/wk) or above was associated with reduced odds of subsequent noninjurious and injurious falls. Participation in brisk walking and both moderate and moderate-vigorous intensity leisure-time physical activity were associated with reduced odds of noninjurious falls.

**Meaning:**

These results support widespread promotion of physical activity, including leisure-time physical activity, to prevent falls with and without injuries in older women.

## Teaser

This cohort study examines whether there are associations between leisure-time physical activity and noninjurious and injurious falls among older women in Australia.

## Introduction

One in three community-dwelling older adults experience at least 1 fall each year. Falls are the world’s second-leading cause of injury mortality.^1^ In Australia, falls account for three-quarters of hospitalized injuries among people aged older than 65 years.^2^ Although some falls do not lead to an injury, people with noninjurious falls can report ongoing fear of falling.^3,4^ Fear of falling impedes independent living, promotes excessive activity avoidance that leads to further decline in physical functioning and leads to a vicious cycle of future falls and fall-related injuries.^4,5,6,7^ To reduce the burden of falls in older adults, better understanding and local and global actions for prevention and management of falls with and without injuries are necessary.^1^

The World Health Organization (WHO) recommends older adults undertake 150 to 300 minutes per week of moderate-intensity physical activity for optimal social, psychological, and physical outcomes, including falls prevention.^8^ Structured exercise, a form of physical activity, conducted in trials reduces falls.^9^ However, inconsistent associations between self-reported physical activity and falls or fall-related injuries have been reported in different meta-analyses of systematic reviews of cohort studies.^10,11,12,13^ One systematic review reported no associations between different levels of physical activity and falls,^10^ whereas another systematic review reported physical activity and reduced falls risk.^11^ Mixed findings have also been reported within other systematic reviews.^12,13^ For instance, a systematic review reported significant reduction of fall-related injury with higher physical activity and insignificant association between higher physical activity and lower rate of any falls.^12^ The associations between physical activity and any falls regardless of injuries and physical activity and falls-related injury could be different. Other factors including study design, sample characteristics, and duration of follow-up could explain the inconsistent findings of the associations between physical activity and falls.^10,12^ The inconsistent results could also be due to heterogeneity of definitions of physical activity participation (eg, physical activity participation during the past year; >3 h/wk of planned exercise) and different cut points used for lower levels of physical activity (eg, <59 min/d; <400 min/d) in included studies in these systematic reviews.^10,12^ Furthermore, definitions of falls used (eg, any fall regardless of injuries; minimum 1 traumatic fall) were also different.^10,12^ With the updated guidelines on physical activity in older adults,^8^ it would be important to understand the associations between the recommended dose of physical activity and falls with and without injuries.

In Australia, the association between physical activity and falls has previously been examined,^14,15,16,17,18,19^ but results are inconclusive. For instance, a longitudinal study reported no associations between high-impact physical activity and incident falls among men aged 70 years and older.^14^ A previous study using data from 1999 and 2002 surveys of the Australian Longitudinal Study on Women’s Health (ALSWH) (cohort born from 1921 to 1926) found that moderate-vigorous leisure-time physical activity (LPA) was associated with reduced risk of falls regardless of injuries, with no association between LPA and injurious falls.^15^ Furthermore, 2 other studies that used data of the cohort born from 1946 to 1951 in the ALSWH showed mixed findings.^16,17^ One study using 2010 and 2013 surveys found no association between LPA and falls regardless of injuries,^16^ whereas a more recent study using data from 2016 and 2019 surveys found associations between LPA and falls with injuries.^17^ Despite the different nature of noninjurious falls and their potential consequences, to our knowledge, previous literature has only investigated any falls (ie, falls regardless of injuries) or injurious falls, and not noninjurious falls.^14,15,16,17,18,19^ Furthermore, to our knowledge, no study has examined and compared the magnitude of the associations of the amounts and types of LPA with noninjurious and injurious falls within the same analysis. Given the limited and inconsistent evidence, we aimed to examine the associations between different amounts and types of LPA and noninjurious and injurious falls.

## Methods

### Data Source

The ALSWH is an ongoing population-based study and has ongoing ethical approval from the human research ethics committees of the Universities of Newcastle and Queensland. It began in 1996 with samples of women born within the years 1921 to 1926, 1946 to 1951, and 1973 to 1978, randomly selected from the government-funded national health insurance database (Medicare), which included all Australian citizens and permanent residents regardless of income.^20,21^ Women from rural and remote areas were intentionally oversampled to ensure good representation of these women.^20^ Data for race and ethnicity were not collected in 1996. There was no exclusion criteria when recruiting participants. Participants provided written informed consent to join and are free to withdraw or suspend their participation at any time.

To understand the associations between LPA and subsequent noninjurious and injurious falls among older people, this study used data from the cohort born from 1946 to 1951 (n = 13 714; aged 45-50 years at baseline) who returned the 2016 survey (for LPA, n = 8622; aged 65-70 years) and 2019 survey (for falls, n = 7956; aged 68-73 years). The cohort born from 1946 to 1951 in the ALSWH was comparable with the corresponding census data, except more women in the ALSWH were employed in 1996.^20^ Most of the participants were born in Australia (70%), reported either married or in a de facto relationship (81%), and were employed (73%) in 1996.^20,22^ This cohort study followed the Strengthening the Reporting of Observational Studies in Epidemiology (STROBE) reporting guideline.^29^

### Exposure: LPA

LPA was measured using a validated version of Active Australia National Physical Activity survey in the 2016 survey.^23^ Participants self-reported the weekly duration of 3 types of LPA: (1) walking briskly (for recreation or exercise or to get from place to place); (2) moderate LPA (eg, social tennis, moderate intensity exercise classes, recreational swimming); and (3) vigorous LPA that makes a person breathe harder or puff and pant (eg, aerobics, vigorous cycling, running, swimming).

Weekly amount of LPA was the sum of total duration of brisk walking, moderate LPA, and vigorous LPA, and was categorized into: (1) 0 minutes, (2) less than 150 minutes, (3) 150 to less than 300 minutes, and (4) greater than or equal to 300 minutes, according to the 150- and 300-minute cut off, which is the recommended targeted duration of moderate-intensity activity by WHO.^8^ Involvement in the aforementioned type of LPA was also dichotomized (yes or no).

### Outcome: Noninjurious and Injurious Falls

Participants in the 2019 survey responded to the following 3 questions about falls in the past 12 months: (1) had a fall to the ground, (2) been injured because of a fall, and (3) sought medical attention for an injury from a fall. Participants were considered to have had a noninjurious fall if they had reported a fall to the ground without any positive response to either the second or third questions, whereas they were considered to have had an injurious fall if they had a positive response to either the second or third question. The outcome was divided into 3 levels: no falls, noninjurious falls, and injurious falls.

### Potential Covariates

Potential confounders were identified using expert knowledge and the literature and incorporated into a directed acyclic graph (DAG), which served as a hypothesized theoretical framework to select a minimally sufficient set of variables confounding the exposure-outcome association. Briefly, a DAG consists of nodes and arrows which show the known or suspected causal relationship between nodes.^24^ We acknowledge the presence of other potential pathways between each node, but the pathways were drawn based on the hypothesized effect of the exposure on the outcome. The DAG was constructed using DAGitty (version 3.0) before performing any analysis (eFigure in Supplement 1).^25,26^ To minimize confounding bias while not introducing other types of bias (eg, overadjustment bias),^27^ the DAG selected the minimally sufficient set of variables to adjust the exposure-outcome association and thus not all the variables within the DAG were required for adjustment.^25,26^ The variables that were identified and included in the final model for adjustment were: age, highest education completed, ability to manage income, Accessibility Remoteness Index of Australia scale (ARIA+),^28^ and housing type.

### Statistical Analysis

Descriptive statistics were used to describe participants’ characteristics. Multinomial logistic regressions were used to examine the association between different amounts and types of LPA and subsequent noninjurious and injurious falls. The reference category in the multinomial logistic regression was no falls. Statistical significance was considered at 2-sided *P* < .05. Statistical analyses were undertaken using SAS version 9.4 (SAS Institute) from September 2022 to February 2023.

## Results

In 1996 when the ALSWH commenced, 13 714 women were recruited. In the 2019 ALSWH survey, of 10 332 participants who were eligible, 7956 (77.0%) completed the survey (Figure). Women with missing exposure in the 2016 survey (544 of 7956 [6.8%]), missing outcome in the 2019 survey (110 of 7956 [1.4%]), missing both exposure and outcome (21 of 7956 [0.3%]), and missing covariates in the 2016 survey (142 of 7956 [1.8%]) were excluded; the final analytical sample size was 7139 (Figure). Characteristics of participants excluded from the analyses due to missing LPA, falls, and/or covariates were similar to those included in the complete case analyses (eTable in Supplement 1). Table 1 presents the characteristics of the included women (mean [SD] age, 67.7 [1.5] years). Most of the women either lived in a major city (2810 [39.4%]) or inner regional areas (2915 [40.8%]) of Australia and had school or higher school certificate (4714 [66.0%]). There were 2410 women (35.2%) who had normal body mass index (BMI is calculated as weight in kilograms divided by height in meters squared; normal BMI is considered in the range of ≥18.5 to <25).

**Figure.**
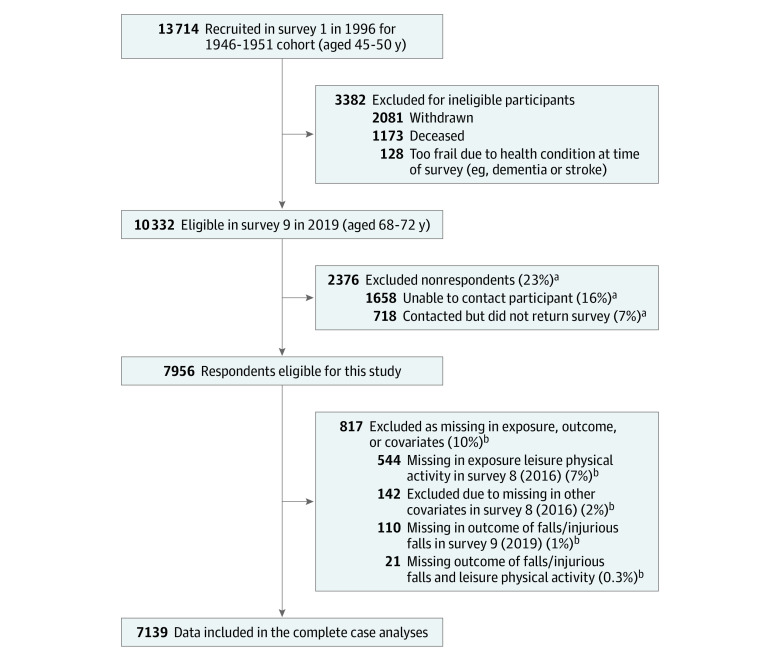
Sample Selection Flowchart ^a^The percentage was calculated based on the number of participants eligible in survey 9 in 2019 (n = 10 332). ^b^The percentage was calculated based on the number of participants eligible for this study (n = 7956).

**Table 1.** Characteristics of Cohort Born From 1946 to 1951 (Survey 8, 2016) in the Australian Longitudinal Study on Women’s Health

Characteristics	Participants, No. (%) (n = 7139)
Age, mean (SD), y^a^^,^^b^	67.7 (1.5)
SF 36 physical health, median (IQR)	85 (70-95)
SF 36 mental Health, median (IQR)	84 (68-92)
Location (ARIA+)^a^	
Major cities of Australia	2810 (39.4)
Inner regional of Australia	2915 (40.8)
Regional (outer regional, remote and very remote Australia)	1414 (19.8)
Housing arrangement^a^	
House/unit/apartment/villa/townhouse	6808 (95.4)
Caravan/mobile home/retirement village/hostel/residential aged care	331 (4.6)
Education^a^	
No formal education	831 (11.6)
School, intermediate, higher school or leaving certificate	4714 (66.0)
University degrees of above	1594 (22.3)
Ability to manage on income^a^	
Impossible	91 (1.3)
Difficult always	506 (7.1)
Difficult sometimes	1388 (19.4)
Not too bad	3500 (49.0)
Easy	1654 (23.2)
BMI^c^	
Underweight	97 (1.4)
Normal	2410 (35.2)
Overweight	2328 (34.0)
Obese	2010 (29.4)
Physical function limitation^d^	1624 (22.9)
Frailty^e^	686 (9.6)

Abbreviations: ARIA+, Accessibility Remoteness Index of Australia scale; BMI, body mass index (calculated as weight in kilograms divided by height in meters squared); SF 36, Short Form 36.

^a^ Variables identified from the directed acyclic graph as potential confounders.

^b^ Participants were recruited by years born and thus age in the cohort ranged from 65 to 70 years. The mean and median age were both 68 years (IQR, 67-69 years).

^c^ Missing data for BMI was n = 294; underweight <18.5; normal ≥18.5 and <25; overweight ≥25 and <30; obese ≥30.

^d^ Women had limitation in physical function if they responded “limited a lot” or “limited a little” in climbing 1 flight of stairs and/or walking 100 meters.

^e^ Women were classified as frail if more than 2 positive responses were recorded out of the 5 components (fatigue, resistance, ambulation, illness, and loss of weight).

Overall, 2012 of 7956 women (28.2%) reported falls in the previous 12 months; 996 had noninjurious falls and 1016 had injurious falls. There were no associations between participation in less than 150 minutes of LPA and noninjurious and injurious falls (Table 2). The reduction in falls associated with 150 to less than 300 minutes of LPA participation was similar for both noninjurious falls (OR, 0.72 [95% CI, 0.58-0.90]) and injurious falls (OR, 0.66 [95% CI, 0.53-0.82]). Participation in greater than or equal to 300 minutes of LPA was associated with a reduction in noninjurious falls (OR, 0.63 [95% CI 0.52-0.77]) and injurious falls (OR, 0.72 [95% CI, 0.60-0.87]). After adjusting for potential confounders, participation in LPA at or above the level recommended by the World Health Organization remained associated with reduced odds of noninjurious falls (150 to <300 minutes: OR, 0.74 [95% CI, 0.59-0.92]; ≥300 minutes: 0.66 [95% CI, 0.54-0.80]) and injurious falls (150 to <300 minutes: OR, 0.70 [95% CI, 0.56-0.88]; ≥300 minutes: OR, 0.77 [95% CI, 0.63-0.93]) (Table 2).

**Table 2.** Amounts and Types of LPA and Subsequent Falls and Injurious Falls (N = 7139)

LPA and duration in the past week	Women, No. (%)			OR (95% CI)			
	Did not fall (n = 5127)	Had noninjurious falls (n = 996)	Had injurious falls (n = 1016)	Crude model^a^		Adjusted model^b^	
				Noninjurious falls	Injurious falls	Noninjurious falls	Injurious falls
Total LPA							
No LPA (n = 1131)	745 (14.5)	194 (19.5)	192 (18.9)	1 [Reference]	1 [Reference]	1 [Reference]	1 [Reference]
1-<150 min (n = 1318)	914 (17.8)	206 (20.7)	198 (19.5)	0.87 (0.70-1.08)	0.84 (0.67-1.05)	0.88 (0.71-1.10)	0.87 (0.70-1.09)
150-<300 min (n = 1458)	1074 (21.0)	202 (20.3)	182 (17.9)	0.72 (0.58-0.90)	0.66 (0.53-0.82)	0.74 (0.59-0.92)	0.70 (0.56-0.88)
≥300 min (n = 3232)	2394 (46.7)	394 (39.6)	444 (43.7)	0.63 (0.52-0.77)	0.72 (0.60-0.87)	0.66 (0.54-0.80)	0.77 (0.63-0.93)
Physical activity that included walking briskly							
No brisk walking reported (n = 1454)	992 (19.4)	230 (23.1)	232 (22.8)	1 [Reference]	1 [Reference]	1 [Reference]	1 [Reference]
Some brisk walking reported (n = 5685)	4135 (80.7)	766 (76.9)	784 (77.2)	0.80 (0.68-0.94)	0.81 (0.69-0.95)	0.83 (0.70-0.97)	0.86 (0.73-1.01)
Physical activity that included moderate leisure activity							
No moderate activity reported (n = 3931)	2771 (54.1)	596 (59.8)	564 (55.5)	1 [Reference]	1 [Reference]	1 [Reference]	1 [Reference]
Some moderate activity reported (n = 3208)	2356 (46.0)	400 (40.2)	452 (44.5)	0.79 (0.69-0.91)	0.94 (0.82-1.08)	0.81 (0.70-0.93)	0.97 (0.84-1.11)
Physical activity that included vigorous leisure activity							
No vigorous activity reported (n = 5074)	3595 (70.1)	731 (73.4)	748 (73.6)	1 [Reference]	1 [Reference]	1 [Reference]	1 [Reference]
Some vigorous activity reported (n = 2065)	1532 (29.9)	265 (26.6)	268 (26.4)	0.85 (0.73-0.99)	0.84 (0.72-0.98)	0.87 (0.74-1.01)	0.86 (0.74-1.01)
Physical activity that included moderate-vigorous leisure activity							
No moderate-vigorous activity reported (n = 5617)	3997 (78.0)	809 (81.2)	811 (79.8)	1 [Reference]	1 [Reference]	1 [Reference]	1 [Reference]
Some moderate-vigorous activity reported (n = 1522)	1130 (22.0)	187 (18.8)	205 (20.2)	0.82 (0.69-0.97)	0.89 (0.76-1.06)	0.84 (0.70-0.99)	0.92 (0.77-1.08)

Abbreviations: LPA, leisure-time physical activity; OR, odds ratio.

^a^ Association between different amount and different types of LPA in survey 8 (2016, when aged 65-70 years) and falls and injurious falls in survey 9 (2019, when aged 68-73 years) were calculated using multinomial logistic regression and presented as OR and 95% CI. Physical activity (ie, different amount of leisure physical activity and whether participated in the different types of LPA) in survey 8 was used as exposure and falls (ie, no falls, noninjurious and injurious falls) in survey 9 as an outcome. “No falls” was used as the reference and “noninjurious and injurious falls” as the alternative outcomes.

^b^ Adjusted model was adjusted for age, Accessibility Remoteness Index of Australia scale, housing arrangement, education, and ability to manage on income.

In unadjusted models, participation in the different types of LPA was associated with lower risk of noninjurious falls, and brisk walking or vigorous LPA participation was associated with lower injurious falls compared with those without LPA (Table 2). After adjusting for potential confounders, those involved in brisk walking (OR, 0.83 [95% CI, 0.70-0.97]), moderate LPA (OR, 0.81 [95% CI, 0.70-0.93]), and moderate-vigorous LPA (OR, 0.84 [95% CI, 0.70-0.99]) had lower odds of noninjurious falls than those who reported no LPA. Participation in vigorous LPA was not associated with noninjurious falls in the adjusted models (OR, 0.87 [95% CI, 0.74-1.01]). Compared with women without LPA, participation in brisk walking (OR, 0.86 [95% CI, 0.73-1.01]) and vigorous LPA (OR, 0.86 [95% CI, 0.74-1.01]) were also not associated with injurious falls in the adjusted models.

## Discussion

This cohort study examined the associations between amounts and types of LPA and subsequent noninjurious and injurious falls in older women. Participation in 150 to less than 300 minutes or greater than or equal to 300 minutes of LPA per week were both associated with lower risk of noninjurious and injurious falls. Brisk walking, moderate LPA, and moderate-vigorous LPA were all associated with lower risk of noninjurious falls.

Participation in LPA at the recommended level (ie, 150 to <300 minutes at moderate-intensity physical activity) by WHO^8^ or above (ie, ≥300 minutes) was associated with lower risk of noninjurious and injurious falls, whereas these associations were not found for those who participated in less than 150 minutes of LPA. A previous population-based study in the United States (n = 102 394) reported lower fall risk regardless of injuries with participation in any LPA among people aged 65 years and older, compared with those who did not engage in any LPA.^30^ Furthermore, another recently published ALSWH study found that only participation in 150 to less than 300 minutes of LPA was associated with reduced injurious falls, but participation of LPA above the recommended level (ie, ≥300 minutes) was not associated with injurious falls.^17^ This previous study did not consider participants who had noninjurious falls. Participants who had falls without injuries were grouped together with participants who did not fall, which means that any differences in factors associated with falls with and without injuries could not be explored.^17^ The consequences of falls without injuries are often neglected, but factors such as fear of falling can increase the risk of future seriously harmful falls.^7^ The present study complements the previous study as we looked at the association of the recommended level of LPA with noninjurious and injurious falls within the same analysis, which has not been previously investigated in Australia. With different statistical methods used in examining the associations between LPA and falls, our study extends the understanding of the associations between LPA and subsequent noninjurious falls, and it affirms the associations between the recommended dose of LPA and both noninjurious and injurious falls.

Interestingly, participation in at least 300 minutes of LPA had a slightly greater reduction in noninjurious than injurious falls. Meanwhile, the reduction in noninjurious and injurious falls was similar for those who participated in 150 to less than 300 minutes of LPA. The exact mechanism of the differences in the reduction in injurious falls between participation in 150 to less than 300 minutes and at least 300 minutes of LPA is unclear, which warrants further investigation. Different dose-response relationships between levels of physical activity and falls regardless of injuries in community-dwelling older adults have previously been reported.^31,32,33^ It is possible that increased falls were due to an increase in exposure to the environment when participating in LPA. Previous cohort studies in China (n = 671, mean age 83 years)^33^ and the US (n = 5995, mean age 74 years)^31^ reported the U-shaped association between physical activity and falls, with increased falls among older people who were more physically active. On the contrary, the Longitudinal Aging Study Amsterdam (n = 1337; mean age 75 years) did not find such U-shaped association between physical activity and falls.^32^ To our knowledge, only the previous ALSWH study of another cohort (ie, 1921-1926 born) had reported the association between LPA participation and falls and LPA and fall-related injuries in separate analyses within the same study.^15^ The previous ALSWH study found reduced falls among those who had a very high level of LPA (ie, ≥40 metabolic equivalents of LPA participation) (OR, 0.67 [95% CI, 0.47-0.95]), but no association was found between LPA and fall-related injuries.^15^ The different findings on LPA and injurious falls between our study and previous ALSWH studies could possibly be explained by the different characteristics of the cohort.^15^ Furthermore, the different statistical methods between the multinomial regression in our study and previous ALSWH studies may have also contributed to the different findings.^15,16^ Our findings indicate the importance of reaching the recommended level of LPA for the prevention of both noninjurious and injurious falls and is consistent with other health benefits associated with being sufficiently active.^8^

Participation in brisk walking, moderate LPA, and moderate-vigorous LPA were all associated with lower risk of subsequent noninjurious falls. A previous population-based study in Australian men (n = 1667; mean age, 77 years) found that swimming was the only activity associated with lower rate of falls.^18^ Gender differences in physical activity participation patterns among older adults have previously been reported.^34^ With the 3 standardized types of activities (ie, walking briskly, moderate, and vigorous-intensity activity) collected in the ALSWH survey, our study extends the understanding of the association between these 3 types of LPA and falls in older women. Meanwhile, the associations found between different types of LPA participation and noninjurious (but not injurious) falls may suggest the distinct associations between LPA and noninjurious falls, and LPA with injurious falls. Furthermore, as trials have previously demonstrated the benefits of specific types of exercise (eg, balance and functional exercises or balance and functional exercises plus resistance exercise) on falls among older adults,^9^ there is potential for an association between combinations of LPA and injurious falls, which warrants future investigation.

The main strength of this study is the large population-based sample of Australian women, which allowed for more precise estimates of the association between LPA and noninjurious and injurious falls. The large sample size also enabled us to have power to analyze both noninjurious and injurious falls, which, to our knowledge, has not been done previously in the literature. Although noninjurious falls can lead to ongoing fear of falling and future seriously harmful falls, noninjurious falls are often neglected.^7^ To our knowledge, this is the first study that examined the association between the recent guideline-recommended amounts of LPA and noninjurious and injurious falls within the same analysis. In addition, the use of DAG to identify confounding variables reduces the overadjustment bias when exploring exposure-outcome association.

### Limitations

There are limitations in our study. LPA was self-reported, and the exact type of the LPA (such as yoga or dance) was not collected. We are unable to identify the nature of the activity that has contributed to the lower risk of noninjurious and injurious falls. Meanwhile, LPA participation could change over time. Another limitation was that the ALSWH is designed to collect a range of health data, but number of falls or severity of the fall-related injuries was not collected. Hence, we were unable to examine the association between LPA and number of falls or severity of the injury. Future studies could use administrative data to examine such associations. Moreover, we are uncertain if the associations found in our study could be generalized to men or people from other countries. Additionally, as with any observational study, it is possible that reverse causality has affected the results (ie, women less prone to fall participated in more LPA). However, we undertook analyses to decrease the risk of this with data on pervious LPA measured in survey 8 and falls measured in survey 9.

## Conclusions

This cohort study examined the WHO-recommended level of LPA and noninjurious and injurious falls. Participation in LPA at the level recommended by WHO or above was associated with lower risk of subsequent noninjurious and injurious falls. Participation in brisk walking, moderate LPA, and moderate-vigorous intensity LPA were also associated with lower risk of noninjurious falls. Our findings suggest support for the widespread promotion of physical activity, including LPA, to reduce the risk of both noninjurious and injurious falls in older women.

## Notes

Supplement 1. **eFigure.** Directed Acyclic Graph (DAG) Showing the Hypothesised Association Between Physical Activity (Exposure) and Falls (Outcome) **eTable.** Characteristics Between Samples Included in the Complete Case Analysis Versus Those Excluded Due to Missing Variables

Supplement 2.
Data Sharing Statement
