# Supplementary material for: Leisure-Time Physical Activity and Falls With and Without Injuries Among Older Adult Women
Source: JAMA Netw Open. 2024 Jan 31;7(1):e2354036. doi: 10.1001/jamanetworkopen.2023.54036 (PMC10831579; doi:10.1001/jamanetworkopen.2023.54036)
Supplement: Supplement 1. — eFigure. Directed Acyclic Graph (DAG) Showing the Hypothesised Association Between Physical Activity (Exposure) and Falls (Outcome) eTable. Characteristics Between Samples Included in the Complete Case Analysis Versus Those Excluded Due to Missing Variables [file jamanetwopen-e2354036-s001.pdf]

Supplemental Online Content

Kwok WS, Khalatbari-Soltani S, Dolja-Gore X, et al. Leisure-time physical activity and falls with and without injuries among older adult women. *JAMA Netw Open*. 2024;7(1):e2354036. doi:10.1001/jamanetworkopen.2023.54036

eFigure. Directed Acyclic Graph (DAG) Showing the Hypothesised Association Between Physical Activity (Exposure) and Falls (Outcome)

eTable. Characteristics Between Samples Included in the Complete Case Analysis Versus Those Excluded Due to Missing Variables

This supplemental material has been provided by the authors to give readers additional information about their work.

© 2024 Kwok et al. *JAMA Network Open.*

eFigure. Directed Acyclic Graph (DAG) Showing The Hypothesised Association Between Physical Activity (Exposure) and Falls (Outcome)

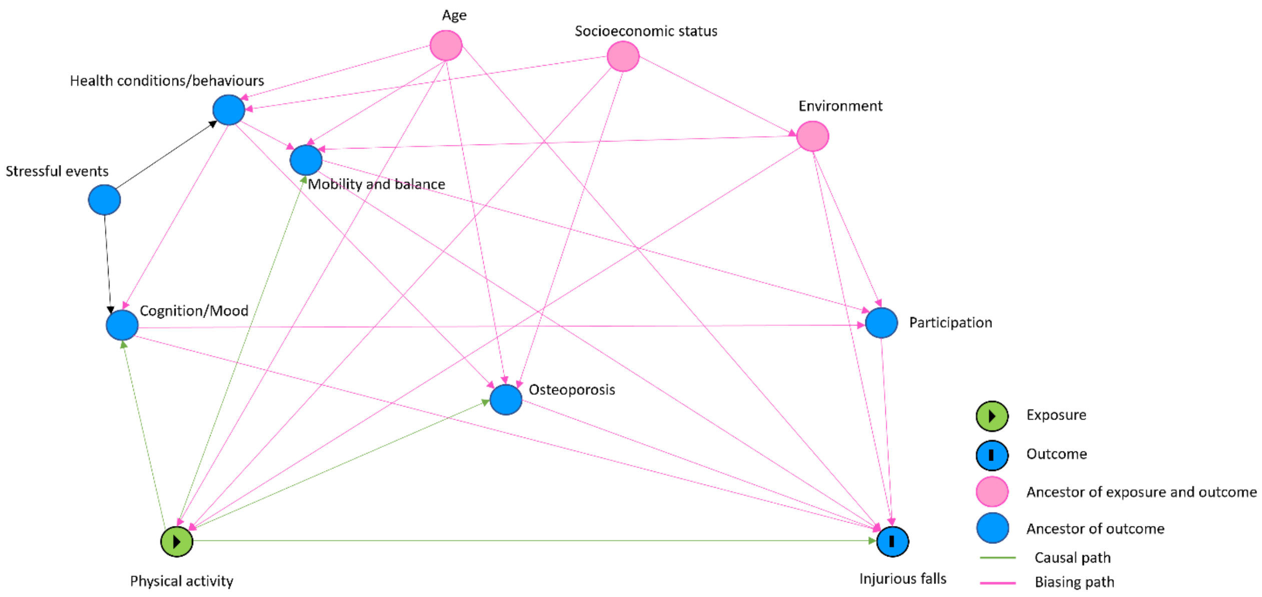

Socioeconomic status was measured using education, and the ability to manage on income; Environment was measured on Accessibility Index of Australia (ARIA+), housing situation. Participation was measured using Short Form 36 (SF36) Social Functioning, SF36 Role – Physical, SF36 Role – Emotion, involvement in volunteer work, taking acre of grandchildren, and providing help to others. Cognition/Mood was measured using SF36 – Mental Health, and have had poor memory. Health conditions/behaviour were measured using SF36 – General Health, number of health conditions, body mass index, alcohol consumption, and smoking. Mobility and balance was measured using SF36 – Physical Functioning, and loss of balance.

eTable. Characteristics Between Samples Included in the Complete Case Analysis Versus Those Excluded Due to Missing Variables

	Participants included n=7,139	Participants excluded[a] n=817
	**mean (SD)**	**mean (SD)**
**Age, years**[b]	68 (1)	68 (1)
**SF 36 Physical Health**, median (IQR)	85 (70 to 95)	85 (65 to 90)
**SF 36 Mental Health**, median (IQR)	84 (68 to 92)	84 (68 to 92)
	**n (%)**	**n (%)**
**Body mass index**[c]		
Underweight	97 (1)	3 (1)
Normal	2,410 (35)	103 (33)
Overweight	2,328 (34)	99 (32)
Obese	2010 (29)	107 (34)
**Location (ARIA+)**[a]		
Major cities of Australia	2,810 (39)	115 (36)
Inner regional of Australia	2,915 (41)	125 (39)
Regional (Outer regional, remote and very remote Australia)	1,414 (20)	77 (24)
**Housing arrangement**		
House/ unit/ apartment/ villa/ townhouse	6,808 (95)	265 (93)
Caravan/ mobile home/ retirement village/ hostel/ residential aged care	331 (5)	20 (7)
**Education**		
No formal education	831 (12)	147 (18)
School, intermediate, higher school or leaving certificate	4,714 (66)	520 (64)
University degrees of above	1,594 (22)	143 (18)
**Ability to manage on income**		
Impossible	91 (1)	6 (2)
Difficult always	506 (7)	27 (9)
Difficult sometimes	1,388 (19)	59 (21)
Not too bad	3,500 (49)	145 (51)
Easy	1,654 (23)	48 (17)
**Physical function limitation**[e]	1,624 (23)	97 (28)
**Frailty**[f]	686 (10)	45 (13)

Characteristics between samples included in the complete case analysis (n=7,139) versus those excluded due to missing variables in leisure physical activity, falls and/or covariates (n=817) of the 1946-51 cohort in the Australian Longitudinal Study on Women's Health.
ARIA+: Accessibility Remoteness Index of Australia scale; IQR: interquartile range; SF 36: Short Form 36

[a] Participants excluded in the study due to either missing exposure, physical activity and or covariates

[b] Participants were recruited by years born and thus age in the cohort ranged between 65 to 70 years. The mean and median age were both 68, IQR 67 to 69 years.

[c] Missing data for participants included n=294; participants excluded n=505.

© 2024 Kwok et al. *JAMA Network Open.*

[d] Missing data for participants excluded: SF36 Physical Health n=465; SF36 Mental health n=467; ARIA+ n=500; housing arrangement n=532; education n=7; ability to manage on income n=532; physical function limitation n=465; frailty n=462.
[e] Women had limitation in physical function if they responded ‘limited a lot’ or ‘limited a little’ in climbing one flight of stairs and/ or walking 100 m.
[f] Women were classified as frail if more than two positive responses were recorded out of the five components (Fatigue, resistance, ambulation, illness and loss of weight).

© 2024 Kwok et al. *JAMA Network Open.*
