# Supplementary material for: Leisure-Time Physical Activity and Falls With and Without Injuries Among Older Adult Women
Source: JAMA Netw Open. 2024 Jan 31;7(1):e2354036. doi: 10.1001/jamanetworkopen.2023.54036 (PMC10831579; doi:10.1001/jamanetworkopen.2023.54036)
Supplement: Supplement 2. — Data Sharing Statement [file jamanetwopen-e2354036-s002.pdf]

Data Sharing Statement

Kwok. Leisure-Time Physical Activity and Falls With and Without Injuries Among Older Adult Women. *JAMA Netw Open*. Published January 31, 2024.
doi:10.1001/jamanetworkopen.2023.54036

Data
Data available: No

Additional Information
Explanation for why data not available: The data that supports the findings of this study is available from the Australian Longitudinal Study on Women's Health (ALSWH). Data in the current study was used following approvals and data sharing agreement and therefore, data is not publicly available. Researchers who wish to access ALSWH data can seek permission from the ALSWH Data Access Committee, https://alswh.org.au/for-data-users/applying-for-data/.
